# Identifying hotspots of cardiometabolic outcomes based on a Bayesian approach: The example of Chile

**DOI:** 10.1371/journal.pone.0235009

**Published:** 2020-06-22

**Authors:** Gloria A. Aguayo, Anna Schritz, Maria Ruiz-Castell, Luis Villarroel, Gonzalo Valdivia, Guy Fagherazzi, Daniel R. Witte, Andrew Lawson

**Affiliations:** 1 Population Health Department, Luxembourg Institute of Health, Strassen, Luxembourg; 2 Competence Center for Methodology and Statistics, Luxembourg Institute of Health, Strassen, Luxembourg; 3 Department of Public Health, School of Medicine, Pontificia Universidad Católica de Chile, Santiago, Chile; 4 Department of Public Health, Aarhus University, Aarhus, Denmark; 5 Danish Diabetes Academy, Odense, Denmark; 6 Department of Public Health Sciences, Medical University of South Carolina, South Carolina, Charleston, United States of America; National Institute of Health and Nutrition, National Institutes of Biomedical Innovation, Health and Nutrition, JAPAN

## Abstract

**Background:**

There is a need to identify priority zones for cardiometabolic prevention. Disease mapping in countries with high heterogeneity in the geographic distribution of the population is challenging. Our goal was to map the cardiometabolic health and identify hotspots of disease using data from a national health survey.

**Methods:**

Using Chile as a case study, we applied a Bayesian hierarchical modelling. We performed a cross-sectional analysis of the 2009–2010 Chilean Health Survey. Outcomes were diabetes (all types), obesity, hypertension, and high LDL cholesterol. To estimate prevalence, we used individual and aggregated data by province. We identified hotspots defined as prevalence in provinces significantly greater than the national prevalence. Models were adjusted for age, sex, their interaction, and sampling weight. We imputed missing data. We applied a joint outcome modelling approach to capture the association between the four outcomes.

**Results:**

We analysed data from 4,780 participants (mean age (SD) 46 (19) years; 60% women). The national prevalence (percentage (95% credible intervals) for diabetes, obesity, hypertension and high LDL cholesterol were 10.9 (4.5, 19.2), 30.0 (17.7, 45.3), 36.4 (16.4, 57.6), and 13.7 (3.4, 32.2) respectively. Prevalence of diabetes was lower in the far south. Prevalence of obesity and hypertension increased from north to far south. Prevalence of high LDL cholesterol was higher in the north and south. A hotspot for diabetes was located in the centre. Hotspots for obesity were mainly situated in the south and far south, for hypertension in the centre, south and far south and for high LDL cholesterol in the far south.

**Conclusions:**

The distribution of cardiometabolic risk factors in Chile has a characteristic pattern with a general trend to a north-south gradient. Our approach is reproducible and demonstrates that the Bayesian approach enables the accurate identification of hotspots and mapping of disease, allowing the identification of areas for cardiometabolic prevention.

## Introduction

Cardiovascular disease is the main cause of death worldwide and also an important cause of disability [1]. Cardiovascular disease and its risk factors have a greater impact on vulnerable populations [2]. Many middle-income countries are undergoing a nutritional transition from a traditional towards a more industrialized diet along with a decline in physical activity [3].

Between 1970 and 1980, Chile experienced a decrease in undernutrition and general mortality and, after 1980, this coincided with an increase in obesity [4]. Data from health surveys in Chile show a growing prevalence of diabetes (4.2%, 9.4%, and 12.3%) and obesity (23.3%, 25.1%, and 34.4%) in 2003, 2009–2010 and 2016–2017 respectively. In contrast, the prevalence of hypertension (33.7% 26.9%, 27.6%) and high cholesterol (35.4%, 38.5%, and 27.8%) have not increased. [5–7].

Chile has an unequal population distribution, having a high population density in the central region and sparsely populated remote areas. The dispersed population presents a challenge to the national public health organization as public hospitals are mostly situated in the central region and remote regions have few hospitals and inhabitants of these areas have difficulty of access to services [8].

Chile is also characterised by heterogeneity in socioeconomic factors such as income and education [9–11]. The northern region has the highest gross domestic product per capita and the south the lowest [8]. In addition, Chile has a mixed private-public health system. Most people with more financial resources are affiliated with the private health system and people with fewer resources are mainly affiliated with the public system. Affiliates in the private system have four times the cost of health care than those in the public health system. Moreover, in the northern region, there are also private hospitals that can be used by people affiliated with the private health system [12].

With limited resources, decision-makers have to prioritize. In order to reduce health disparities, public health policy makers require detailed regional and local knowledge about the distribution of chronic diseases and their risk factors to better allocate resources to meet population need [13]. However, in many countries, only national or regional health data are available and it is necessary to analyse the data in a smaller geographic unit to have more refined data.

In addition to traditional determinants of health for chronic diseases, such as demographic characteristics and behavioural factors, neighbourhood can influence health [14]. De Groot (2019) show that populations in urban areas had higher levels of low-density lipoprotein (LDL) cholesterol and triglycerides compared to rural areas [15]. In addition, differences in neighbourhoods in terms of food accessibility and walkability may be associated with higher cardiovascular mortality and premature death [16].

Moreover, national prevalence statistics may not reveal differences between regions or geographic inequalities [17]. Traditional analytic approaches, such as those used in population-based surveys -designed for national level inferences- often lack statistical power to explore sparsely populated geographical areas, unless these are consciously oversampled [18]. Bayesian analyses can be used to improve prevalence estimates in sparsely sampled areas by inferring information from surrounding and similar areas.

The main cause of mortality in the Chilean adult population are circulatory system diseases [19]. Moreover, Chile has a high prevalence of diabetes, obesity and hypertension. Therefore, in this study, we aim to identify hotspots of cardiometabolic risk factors to detect key areas where public intervention is needed. In addition, we aim to examine the geographical variation in the prevalence of cardiometabolic health using a Bayesian hierarchical modelling. Our hypothesis is that we will observe areas with a high prevalence of cardiometabolic conditions and that we will observe a geographic trend in the distribution of health conditions.

## Methods

### Study population/design

The study population was composed of participants in the Chilean Health Survey. This survey is a population-based study representative of the Chilean adult population that collects data on demographic, behavioural, physical and mental health. It has a clinical examination with blood samples, and individual data about place of residence (region, province and commune). The survey has a cross-sectional design and data collection has been carried out in three waves to date (2003, 2009–2010 and 2016–2017) [5–7]. It applies a methodology comparable with other health surveys in the Americas [20].

We analyse the second wave of the Chilean Health Survey 2009–2010 (CHS-2), because this was the first time that all regions of the country were sampled and resident location data were collected and available for research at the time of the analysis. The sample was random with households as units. The national, regional, urban and rural levels were represented in the design. The sample was complex and obtained through a stratified and multistage sampling process and with non-proportional distribution of surveys by stratum [6]. The target population was participants aged 15 years and older. According to a projection of population census data from 2002 to January 2010, the total population of 15 years and over was 13,177,032 inhabitants. The survey was answered by 85% of the eligible population and 5,434 people were finally interviewed. The Research Ethics Committee of the School of Medicine of the Pontificia Universidad Católica de Chile gave the Ethical approval and all participants signed an informed consent.

We included people who participated in all CHS-2 visits: survey, clinical examination with blood samples and provided geographic data.

For organizational purposes, the Chilean territory is divided into three hierarchical units: regions, provinces and municipalities. The CHS-2 was the first wave of the Chilean Health Survey to sample participants in all regions of the country. In order to have a detailed and at the same time interpretable information, provinces were chosen as the unit of analysis. Additionally, for description purposes, we described greater geographic units called great regions and divided the country in north (latitude: -18° to -31°), centre (latitude: -32° to -37°), south (latitude: -38° to -43°) and far south great region (latitude: -44° to -53°). For creating the posterior mean prevalence maps, we used a basic map of Chile which is freely available at: http://labgeo.ufro.cl/catalogos/chile.html [21]

### Statistical analyses

We calculated descriptive statistics using the unweighted sample population, comparing characteristics by sex and geographic area.

#### Outcomes

Diabetes was diagnosed with a fasting glucose ≥ 126 mg/dl or with self-reported medical diagnosis (excluding diabetes during pregnancy) [22]. Obesity was diagnosed with a BMI ≥ 30 kg/m^2^ calculated with measured weight and height [23]. Hypertension was diagnosed with either a measured systolic blood pressure ≥ 140 mmHg or diastolic blood pressure ≥ 90 mmHg or self- reported antihypertensive treatment [24]. High LDL was diagnosed with a value > 160 mg/dl [25].

#### Descriptive variables

Continuous variables are described as mean (SD) or median (IQR) according to the observed distribution and calculated from non-weighted sample population. Urban regions were defined as group of concentrated dwellings with more than 2,000 inhabitants. Education and income were used as proxies of the socioeconomic status [9, 11]. Education level was categorized as low (< 8 years), intermediate (8 to 12 years) and high (> 12 years). Income was categorized into tertiles in low (<254€/month), intermediate (254–491€/month) and high income (>491€/month) Physical activity was defined as self-reported frequency of at least once a week of mild, moderate or vigorous activity. Underweight was defined as BMI < 18.5; normal weight as 18.5 ≤ BMI < 25; overweight 25 ≤ BMI < 30; and obesity as BMI ≥ 30 kg/m^2^. Central obesity was defined as waist circumference > 102 cm in men or > 88 cm in women. Consumption of alcohol was classified as usual consumption of 3 or more drinks a day, 2 drinks a day, 1 drink a day, no drink a day or never.

#### Bayesian estimation for handling missing data

To deal with missing data we applied Bayesian imputation. All imputation in Bayesian models was done within Markov chain Monte Carlo. We assumed a missing at random mechanism. We observed missing data in determinants and outcomes, therefore we applied a Bayesian paradigm (BUGS software) for imputation of missing data in outcomes [26]. In addition, we specified priors for missing data in determinants. In the case of missing sampling for some provinces, we assumed restricted prior distributions using the global mean sampling weight.

#### Bayesian hierarchical modelling for estimating probabilities

The statistical methods of this study are explained in detail in Lawson et al [27]. To study the geographic distribution of outcomes and their interrelations, we chose a flexible Bayesian hierarchical modelling approach, which included extra georeferenced confounding [28]. Models took into account the individual and aggregated (province) dimensions.

At the individual level, our models included fixed and random effects. The random effects consisted of uncorrelated and correlated spatial effects. The model included a sampling weight for each individual, as well as age, sex and age-sex interaction as fixed effects [29, 30].

Additionally, we fitted a model at an aggregated level (by province). We divided the sum of cases by condition and province by the number of samples per province. The fixed effects were the mean sampling weight per province, the mean age per province, the percentage of males per province and the age-sex interaction. We used the aggregated model for calculating the posterior mean prevalence. We report posterior mean prevalence with 95% credible intervals (95% CrI), which correspond to the 2.5 and 97.5 percentiles of the posterior mean distribution. We included spatial effects in the aggregated model with the objective of representing the province within which the individual resides. These spatial effects included an uncorrelated effect, which captures the clustering tendency of the outcome and deals with small number of sampling in some provinces [29].

We applied a joint model approach assuming a correlation between each outcome (diabetes, obesity, hypertension and high LDL cholesterol) within the same individual [31]. To estimate overall posterior mean probabilities, we calculated the average of the global simulated parameters of all provinces with 95% CrI based on quantile probability intervals. The two Bayesian models described in this analysis (imputation model, individual and aggregated by province joint outcome models) are different but they were run in the same Markov Chain Monte Carlo iterations. The equations / terms of the statistical analysis model have been published by us elsewhere [32]

To roughly compare Bayesian hierarchical modelling with a frequentist approach to estimate the prevalence of the disease, we fitted generalized linear mixed-effects models using penalized quasi-likelihood separated for each outcome. Fixed effects included age, sex, age-sex interaction and sampling weights. Random effects were provinces. The results were parameter estimates and their 95% confidence intervals.

#### Hotspots of cardiometabolic health

To detect provinces with exceptionally high prevalence of cardiovascular risk factors and diabetes, we used posterior exceedance probability estimates that were greater than a chosen threshold [33]. The chosen thresholds were the estimated median values of the posterior prevalence for each outcome.

### Sensitivity analysis

We adjusted the basic model for income and education at individual level in order to assess the impact of these confounders.

We used WinBUGS [34] to fit joint models, R2WinBUGS and rube packages [35] to call a BUGS model, tmap package [36] for creating maps and package MASS [37] to fit generalized linear mixed models via penalized quasi-likelihood.

## Results

### Descriptive statistics

Among the 5,434 participants included in the CHS-2, 5,293 (97.4%) also had residential location data. From those, 4,780 participated in the physical examination/blood sampling and were included in this analysis. Sixty per cent of participants were women and the mean age (SD) was 46 years (18.5).

Table 1 shows demographic and lifestyle characteristics of the study population stratified by sex and geographic area (great regions). The last column shows the percentage of missing data for each variable. All variables had less than 5% missing data except LDL cholesterol that had 44%. The percentage or urban people was significantly higher in the north and lower in the south compared to the centre. In addition, a significant lower level of education, income and percentage of employment was observed in the south compared to the centre. In contrast, high to moderate physical activity level and percentage of non-smokers was higher in the south compared to the centre. A higher income level was observed in the north compared to the centre.

**Table 1:** Characteristics of the sample study (Chilean Health Survey 2009–2010, n = 4,780) by sex and geographic area^a^.

	All	Men	Women	North	Centre	South	Far South	Missing
Variables	(n = 4,780)	(n = 1,915)	(n = 2,865)	(n = 1,410)	(n = 1,974)	(n = 569)	(n = 827)	(%)
Age (years), mean (SD)	46 (19)	46 (18)	47 (19)	45 (19)	46 (19)	48 (19)^b^	47 (18)	0
Urban region, n (%)	4,073 (85)	1,644 (86)	2,429 (85)	1,311 (93)^b^	1,678(85)	397 (70)^b^	687 (83)	0
Education, n (%)								0.4
< 8 years	1,262 (27)	462 (24)	800 (28)^b^	292 (21)^b^	516 (26)	212 (38)^b^	242 (29)	
8–12 years	2,606 (55)	1,084 (57)	1,522 (53)^b^	825 (59)^b^	1,045 (53)	290 (51)^b^	446 (54)	
> 12 years	895 (19)	364 (19)	531 (19)	287 (20)^b^	409 (21)	64 (11)^b^	135 (16)^b^	
Paid work, n (%)	2,186 (46)	1,231 (65)	955 (34)^b^	641 (46)	909 (46)	218 (39)^b^	418 (51)^b^	0.9
Income, n (%)								8.5
< 278 USD month	1,755 (40)	588 (34)	1,167 (44)^b^	436 (34)^b^	698 (39)	316 (58)^b^	305 (40)	
278–539 USD month	1,461 (33)	608 (35)	853 (32)	435 (34)	629 (35)	148 (27)^b^	249 (33)	
> 539 USD month	525 (26)	525 (31)	631 (24)^b^	402 (32)^b^	464 (26)	77 (14)^b^	213 (28)	
Physical activity level^c^, n (%)								2.3
Low	1,502 (32)	504 (27)	998 (36)^b^	493 (36)^b^	628 (33)	113 (20)^b^	268 (34)	
Moderate	923 (20)	307 (17)	616 (22)	292 (21)	391 (20)	88 (16)^b^	152 (19)	
High	2,246 (48)	1,052 (57)	1,194) 43^b^	594 (43)^b^	912 (47)	361 (64)^b^	379 (47)	
Smoking status, n (%)								2.5
Current	1,666 (36)	729 (39)	937 (34)^b^	497 (36)^b^	715 (37)	152 (27)^b^	302 (38)	
Former	1,049 (23)	511 (27)	538 (19)^b^	275 (20)^b^	468 (24)	126 (22)^b^	180 (23)	
Never	1,947 (42)	625 (34)	1322 (47)^b^	598 (44)^b^	750 (39)	286 (51)^b^	313 (39)	
Alcohol, n (%)								0.8
≥ 3 drinks a day	1276 (27)	891 (47)	385 (14)^b^	412 (29)^b^	515 (26)	143 (25)	206 (25)	
2 drinks a day	754 (16)	339 (18)	415 (17)^b^	234 (17)	308 (16)	77 (14)	135 (17)	
1 drinks a day	1264 (27)	309 (16)	955 (34)^b^	377 (27)	551 (28)	131 (23)	205 (25)	
0 drinks a day	1450 (31)	360 (19)	1090 (38)^b^	382 (27)	582 (30)	215 (38)^b^	271 (33)^b^	
BMI, kg/m^2^, mean (SD)	28 (5)	27 (5)	28 (6)	28 (5)	28 (6)	28 (5)^b^	29 (5)^b^	1.5
Nutritional status^d^, n (%)								1.5
Obesity	1373 (29)	444 (24)	929 (33)^b^	372 (27)	522 (27)	197 (35)^b^	282 (34)^b^	
Overweight	1884 (40)	861 (46)	1023 (36)^b^	588 (42)^b^	782 (40)	197 (35)	317 (39)	
Normal weight	1371 (29)	563 (30)	808 (29)^b^	397 (29)	601 (31)	160 (29)^b^	213 (26)^b^	
Underweight	79 (1.7)	22 (1.2)	57 (2.0)	35 (2.5)	32 (1.7)	5 (0.9)	7 (0.9)	
Central obesity^e^, n (%)	2,022 (43)	457 (24)	1,565 (55)^b^	585 (42)	777 (40)	280 (50)^b^	380 (46)^b^	0.9
Glycaemia (mg/dl), median (IQR)	89 (83, 97)	91 (85, 99)	88 (82, 96)^b^	90 (84, 98)	89 (83, 97)	90 (84, 99)	89 (83, 96)	3.2
Diabetes^f^, n (%)	506 (11)	200 (10)	306 (11)	153 (11)	211 (11)	68 (12)	68 (9)	0
SBP, mm Hg, mean (SD)	127 (23)	132 (22)^b^	124 (23)^b^	125 (22)^b^	128 (22)	131 (25)^b^	129 (22)	0.9
DBP, mm Hg, mean (SD)	76 (11)	79 (12)^b^	74 (11)^b^	75 (12)	76 (11)	77 (12)	77 (11)	0.9
Hypertension^g^, n (%)	1685 (36)	709 (37)	976 (34)^b^	424 (30)^b^	733 (37)	230 (41)	298 (36)	0.6
High LDL^h^, n (%)	315 (11.7)	134 (12.1)	181 (11.4)	99 (12.7)^b^	99 (9.1)	37 (11.3)	80 (16.2)^b^	44.0
Myocardial infarction (%)^i^	182 (3.8)	88 (4.6)	94 (3.3)	58 (4.2)	73 (3.7)	22 (3.9)	29 (3.5)	3.5
Stroke, n (%)^i^	134 (2.8)	60 (3.1)	74 (2.6)	35 (2.5)	60 (3.0)	21 (3.7)	18 (2.29)	0.4

Abbreviations: SBP: systolic blood pressure; DBP: diastolic blood pressure.

^a^ Mean (SD), median (IQR) or n (%) calculated from the non-weighted sample population.

^b^P < 0.05 (linear regression for means, Kruskal-Wallis test for medians and multinomial logistic regression for proportions) for comparison between sex (men is the reference) and region categories (centre is the reference).

^c^ Self-reported frequency of at least once a week of mild/moderate/vigorous activity.

^d^Underweight: BMI < 18.5; normal weight: BMI ≥ 18.5 and < 25; overweight BMI ≥ 25 and < 30; obesity: BMI ≥ 30 kg/m^2^.

^e^Central obesity defined as waist circumference > 102 cm in men or > 88 cm in women.

^f^ Diabetes defined as self-reported medical diagnosis or glycaemia ≥ 126 mg/dl (≥7 mmol/L).

^g^ Hypertension defined as systolic ≥ 140 or diastolic blood pressure ≥ 90 mm Hg or taking antihypertensive medication.

^h^ High LDL defined as LDL cholesterol> 160 mg/dl.

^i^ Self-reported medical diagnosis.

### Joint modelling outcome: Posterior mean prevalence (Bayesian prevalence)

The nationwide posterior mean prevalence (% (95% CrI)) of diabetes, obesity, hypertension and high LDL cholesterol were 10.9 (4.5, 19.2), 30.0 (17.7, 45.3), 36.4 (16.4, 57.6), and 13.7 (3.4, 32.2) respectively.

**Fig 1:**
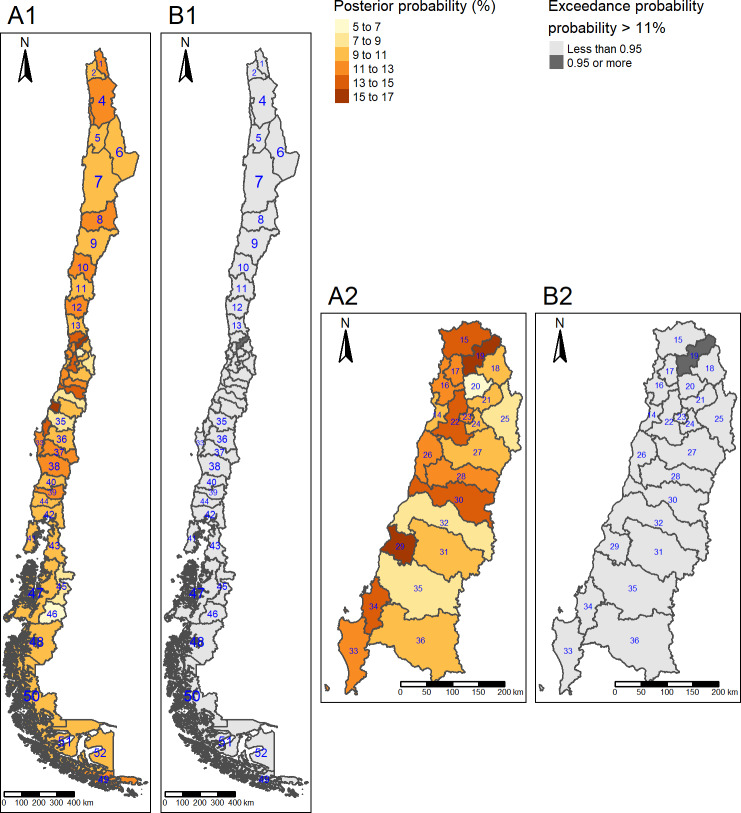
Diabetes posterior mean prevalence and hotspots. (A1) Posterior mean prevalence, all areas. (A2) Posterior mean prevalence, Central area. Colours are in increasing gradient for prevalence (light yellow 5–7% to dark red 15–17% of diabetes prevalence). (B1) Hotspots, All areas. (B2) Hotspots, Central areas. The hotspot is shown in dark grey (exceedance probability significant for ≥ 11%). North: 1 = Parinacota; 2 = Arica; 3 = Iquique; 4 = Tamarugal; 5 = Tocopilla; 6 = El Loa; 7 = Antofagasta; 8 = Chañaral; 9 = Copiapó; 10 = Huasco; 11 = Elqui; 12 = Limarí; 13 = Choapa; Centre: 14 = San Antonio; 15 = Petorca; 16 = Valparaíso; 17 = Quillota; 18 = Los Andes; 19 = San Felipe; 20 = Chacabuco; 21 = Santiago; 22 = Melipilla; 23 = Talagante; 24 = Maipo; 25 = Cordillera; 26 = Cardenal Caro; 27 = Cachapoal; 28 = Colchagua; 29 = Cauquenes; 30 = Curicó; 31 = Linares; 32 = Talca; 33 = Arauco; 34 = Concepción; 35 = Ñuble; 36 = Biobio; South: 37 = Malleco; 38 = Cautín; 39 = Ranco; 40 = Valdivia; 41 = Chiloé; 42 = Llanquihue; 43 = Palena; 44 = Osorno; Far South: 45 = Coyhaique; 46 = General Carrera; 47 = Aisén; 48 = Capitan Prat; 49 = Antártica Chilena; 50 = Última Esperanza; 51 = Magallanes; 52 = Tierra del Fuego.Republished from http://labgeo.ufro.cl/ under a CC BY license, with permission from C. Albers, original copyright 2020.

**Fig 2:**
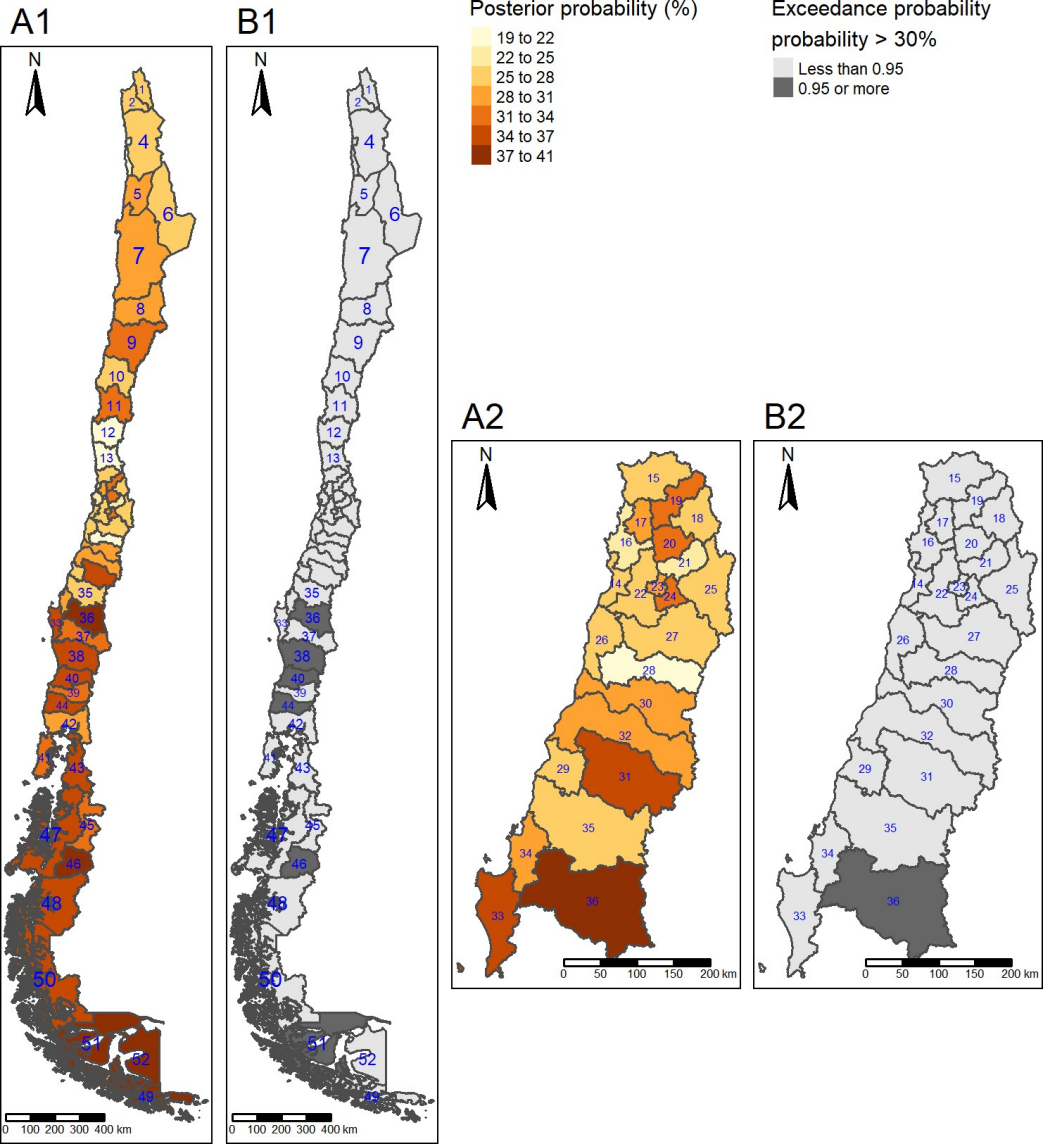
Obesity posterior mean prevalence and hotspots. (A1) Posterior mean prevalence, all areas. (A2) Posterior mean prevalence, Central area. Colours are in increasing gradient for prevalence (light yellow 19–22% to dark red 37–41% of obesity prevalence). (B1) Hotspots, All areas. (B2) Hotspots, Central areas. Hotspots are shown in dark grey (exceedance probability significant for ≥ 30%). North: 1 = Parinacota; 2 = Arica; 3 = Iquique; 4 = Tamarugal; 5 = Tocopilla; 6 = El Loa; 7 = Antofagasta; 8 = Chañaral; 9 = Copiapó; 10 = Huasco; 11 = Elqui; 12 = Limarí; 13 = Choapa; Centre: 14 = San Antonio; 15 = Petorca; 16 = Valparaíso; 17 = Quillota; 18 = Los Andes; 19 = San Felipe; 20 = Chacabuco; 21 = Santiago; 22 = Melipilla; 23 = Talagante; 24 = Maipo; 25 = Cordillera; 26 = Cardenal Caro; 27 = Cachapoal; 28 = Colchagua; 29 = Cauquenes; 30 = Curicó; 31 = Linares; 32 = Talca; 33 = Arauco; 34 = Concepción; 35 = Ñuble; 36 = Biobio; South: 37 = Malleco; 38 = Cautín; 39 = Ranco; 40 = Valdivia; 41 = Chiloé; 42 = Llanquihue; 43 = Palena; 44 = Osorno; Far South: 45 = Coyhaique; 46 = General Carrera; 47 = Aisén; 48 = Capitan Prat; 49 = Antártica Chilena; 50 = Última Esperanza; 51 = Magallanes; 52 = Tierra del Fuego.Republished from http://labgeo.ufro.cl/ under a CC BY license, with permission from C. Albers, original copyright 2020.

**Fig 3:**
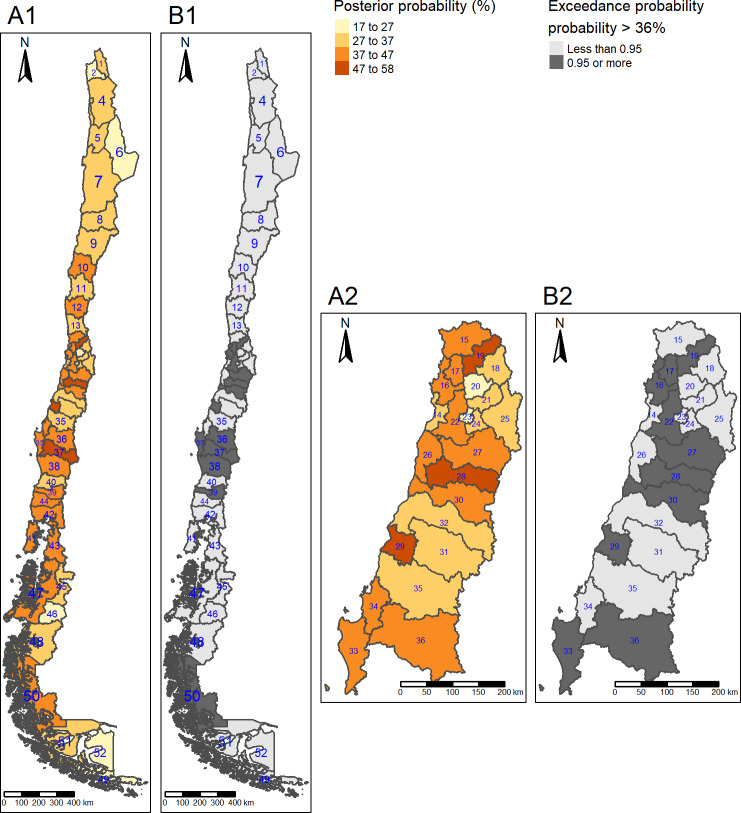
Hypertension posterior mean prevalence and hotspots. (A1) Posterior mean prevalence, all areas. (A2) Posterior mean prevalence, Central area. Colours are in increasing gradient for prevalence (light yellow 17–27% to dark red 47–58% of hypertension prevalence). (B1) Hotspots, All areas. (B2) Hotspots, Central areas. Hotspots are shown in dark grey (exceedance probability significant for ≥ 36%). North: 1 = Parinacota; 2 = Arica; 3 = Iquique; 4 = Tamarugal; 5 = Tocopilla; 6 = El Loa; 7 = Antofagasta; 8 = Chañaral; 9 = Copiapó; 10 = Huasco; 11 = Elqui; 12 = Limarí; 13 = Choapa; Centre: 14 = San Antonio; 15 = Petorca; 16 = Valparaíso; 17 = Quillota; 18 = Los Andes; 19 = San Felipe; 20 = Chacabuco; 21 = Santiago; 22 = Melipilla; 23 = Talagante; 24 = Maipo; 25 = Cordillera; 26 = Cardenal Caro; 27 = Cachapoal; 28 = Colchagua; 29 = Cauquenes; 30 = Curicó; 31 = Linares; 32 = Talca; 33 = Arauco; 34 = Concepción; 35 = Ñuble; 36 = Biobio; South: 37 = Malleco; 38 = Cautín; 39 = Ranco; 40 = Valdivia; 41 = Chiloé; 42 = Llanquihue; 43 = Palena; 44 = Osorno; Far South: 45 = Coyhaique; 46 = General Carrera; 47 = Aisén; 48 = Capitan Prat; 49 = Antártica Chilena; 50 = Última Esperanza; 51 = Magallanes; 52 = Tierra del Fuego. Republished from http://labgeo.ufro.cl/ under a CC BY license, with permission from C. Albers, original copyright 2020.

**Fig 4:**
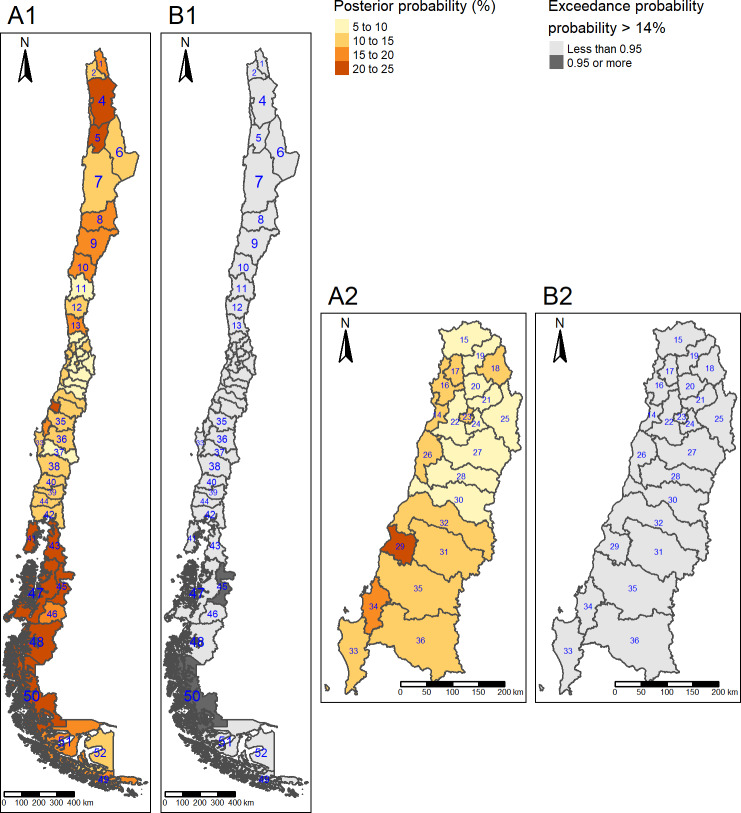
High LDL cholesterol posterior mean prevalence and hotspots. (A1) Posterior mean prevalence, all areas. (A2) Posterior mean prevalence, Central area. Colours are in increasing gradient for prevalence (light yellow 5–10% to dark red 20–25% of high LDL cholesterol prevalence). (B1) Hotspots, All areas. (B2) Hotspots, Central areas. Hotspots are shown in dark grey (exceedance probability significant for ≥ 14%). North: 1 = Parinacota; 2 = Arica; 3 = Iquique; 4 = Tamarugal; 5 = Tocopilla; 6 = El Loa; 7 = Antofagasta; 8 = Chañaral; 9 = Copiapó; 10 = Huasco; 11 = Elqui; 12 = Limarí; 13 = Choapa; Centre: 14 = San Antonio; 15 = Petorca; 16 = Valparaíso; 17 = Quillota; 18 = Los Andes; 19 = San Felipe; 20 = Chacabuco; 21 = Santiago; 22 = Melipilla; 23 = Talagante; 24 = Maipo; 25 = Cordillera; 26 = Cardenal Caro; 27 = Cachapoal; 28 = Colchagua; 29 = Cauquenes; 30 = Curicó; 31 = Linares; 32 = Talca; 33 = Arauco; 34 = Concepción; 35 = Ñuble; 36 = Biobio; South: 37 = Malleco; 38 = Cautín; 39 = Ranco; 40 = Valdivia; 41 = Chiloé; 42 = Llanquihue; 43 = Palena; 44 = Osorno; Far South: 45 = Coyhaique; 46 = General Carrera; 47 = Aisén; 48 = Capitan Prat; 49 = Antártica Chilena; 50 = Última Esperanza; 51 = Magallanes; 52 = Tierra del Fuego. Republished from http://labgeo.ufro.cl/ under a CC BY license, with permission from C. Albers, original copyright 2020.

Posterior mean prevalence of diabetes was lower in the far south (9.0%) compared to the central, region (11%). Posterior mean prevalence of obesity was higher in the south (34%) and far south (37%), compared to the centre (28%). Posterior mean prevalence of hypertension was higher in the south (42%) compared to the centre (39%). Posterior mean prevalence of high LDL cholesterol was higher in the north (15%), south (15%) and far south (19%) (Figs 1–4) compared to the centre (11%).

The highest posterior mean prevalence of diabetes was observed in three provinces located in the centre region: Cauquenes (province number (pn) 29, 16% (95% CrI 9.8, 24)), San Felipe de Aconcagua (pn 19, 16% (95% CrI 11, 24)) and Melipilla (pn 22, 14% (95% CrI 9.3, 22)) (Fig 1A1–1A2).

The highest posterior mean prevalence of obesity was observed in three provinces located in the far south region: Antártica (pn 49, 41% (95% CrI 14, 71)), Tierra del Fuego (pn 52, 40% (95% CrI 19, 63)) and Magallanes (pn 51, 38% (95% CrI 33, 43)) (Fig 2A1–2A2).

The highest posterior mean prevalence of hypertension was observed in Cauquenes (centre, pn 29, 57% (95% CrI 46; 67), Malleco (south, pn 37, 56% (95% CrI 47, 64)) and Colchagua (centre, pn 28, 54% (95% CrI 48, 61)) (Fig 3A1–3A2).

The highest posterior mean prevalence of high LDL cholesterol was observed in three provinces located in the far south region: Última Esperanza (pn 47, 24% (95% CrI 13, 37)), Capitán Prat (pn 48, 22% (95% CrI 3.0, 63)) and Coyhaique (pn 45, 22% (95% CrI 14, 32)) (Fig 4A1–4A2).

S1 Table shows the frequentist prevalence of cardiometabolic outcomes for each province. In contrast to the Bayesian hierarchical modelling, it was not possible to calculate the prevalence for non-sampled provinces.

In Bayesian hierarchical individual level modelling, older age was associated with a higher risk for all outcomes (diabetes: coefficient 0.06 (95% CrI 0.05, 0.07); obesity: coefficient 0.02 (95% CrI 0.01, 0.02); hypertension: coefficient 0.12 (95% CrI 0.11, 0.13); high LDL cholesterol: coefficient 0.04 (95% CrI 0.03, 0.05)). Male was associated with a lower risk for obesity (coefficient -0.54 (95% CrI -0.70, -0.38)) and higher for hypertension (coefficient 0.40 (95% CrI 0.20, 0.59)). The interaction male-age was significant and positive for obesity (coefficient 0.357 (95% CrI 0.065, 0.64)), which means that the observed effect of lower risk of male for obesity decreases with age.

In the sensitivity analysis where the basic models were further adjusted by income and education, there was no significant changes in prevalence of the outcomes (S2 Table). Regarding estimation, we did not observe any significant associations of education or income at the aggregate level, although at the individual level, a significant association of education for obesity (mean estimation (95% CrI) 0.31 (0.08, 0.54) and income for hypertension was observed (mean estimation (95% CrI) -0.019 (-0.032, -0.006)).

### Joint modelling outcome: Hotspots

For diabetes, we detected only one hotspot: San Felipe de Aconcagua (pn 19), situated in the centre great region (Fig 1B1–1B2).

For obesity, we detected one hotspot situated in the centre (Biobío (pn 43)), three hotspots situated in provinces in the south (Cautín (pn 38), Valdivia (pn 40), Osorno (pn 44)) and two situated in the far south (Magallanes (pn 51) and General Carrera (pn 46)) (Fig 2B1–2B2).

For hypertension, we detected hotspots mostly located in the central region of Chile (10 hotspots: Colchagua (pn28), San Felipe de Aconcagua (pn19), Cauquenes (pn 29), Valparaíso (pn 16), Biobío (pn 36), Cachapoal (pn 27), Quillota (pn 17), Curicó (pn 30), Melipilla (pn 22) and Arauco (pn 33)). There were two hotspots in the south (Malleco (pn37) and Ranco (pn39) and one in the far south (Última Esperanza (pn 50) (Fig 3B1–3B2).

The hotspots for high LDL were located in the far south (Coyhaique (pn 45) and Última Esperanza (pn 50) (Fig 4B1–4B2).

## Discussion

The results of this study revealed cardiometabolic health hotspots mainly in the centre, south and far south great regions. We observed also a characteristic pattern of chronic diseases in the Chilean territory, with increasing prevalence from north to south and hot spots mainly in the central and southern regions. Our results showed that Chile is one of the countries with the highest prevalence of diabetes and obesity in the Americas [38, 39]. The prevalence of hypertension in Chile was higher than the global age-standardized prevalence in Latin America [40]. In contrast, the prevalence of high LDL cholesterol was lower than the prevalence in the US in 2010 [41].

Hotspots were often located in more deprived areas. For example, a diabetes hotspot was located in San Felipe de los Andes, which was in the 4th percentile of the Socio-Economic Development Index (SEDI), which included income per capita, education and housing) and two obesity hotspots were located in Biobío and Cautín, which were at the 1st percentile of SEDI, the lowest in the country [42]. These findings could be attributed to environmental factors present in clusters, such as a more accelerated nutritional transition [43], as described in the provinces in the centre area in the Maule region (provinces 29 to 32). In addition, the socio-economic level of the central area was very heterogeneous. The highest prevalence of diabetes and obesity was Cauquenes (pn 29), in the region of Maule, which has one of the lowest gross domestic product per capita in the country [8]. In addition, we found that some socioeconomic factors such as education or income were potential confounder factors (S2 Table).

Differences in the prevalence of chronic diseases among different regions may reflect geographic inequities, such as adequate and timely access to health facilities in remote areas. Research in Russia has shown that rapid access to percutaneous coronary intervention is highly dependent on the region and more difficult for rural areas [13]. In Chile, access to health facilities could be particularly difficult in rural areas in the south and far south.

Our finding, that diabetes was more prevalent in the central and southern region, could be explained by certain socio-economic characteristics such as low income/education, as is demonstrated in other surveys [44]. However, the high prevalence of diabetes in the central region could also be attributed to a combination of factors other than income and education, such as high prevalence of obesity, high blood pressure and lower physical activity levels [45]. In addition, environmental factors can be a possible cause for higher diabetes prevalence such as higher rates of urbanization [46].

Obesity was more prevalent in the south and far south of Chile. We found a significant association of low compared to high income. In addition, the south is a region with lower socio-economic resources, which could partly explain the high prevalence of obesity through food choice [47]. In the far south, the climate and geographic remoteness with low availability of healthy food could influence the high prevalence of obesity. Similar geographic conditions with the same problems of food availability can be observed in the far north of Canada [48].

Hypertension was less prevalent in the north and more prevalent in the south. We observed a significant association of income level on hypertension. Therefore, differences in income, education and nutrition habits can explain, at least in part, our results. The north and south have the highest and lowest income levels respectively and at the same time, the lowest and highest hypertension prevalence. Our results are in agreement with a meta-analysis including 54 studies that found that lower socioeconomic status, and especially lower education was associated with higher blood pressure [49]. High LDL cholesterol was less prevalent in the centre of Chile. These results suggest the possible role of the high concentration of health facilities in the centre of Chile.

Traditional generalized models that assume independence of observations are not the most appropriate method to analyse spatial data, because they ignore the spatial autocorrelation between people living in the same area [18]. Rather than calculating the prevalence of a condition in a region by using only data from that region, Bayesian hierarchical modelling use all available data, along with the geographical structure to obtain the best possible estimate of disease prevalence for a given area. These models infer information from surrounding and similar areas to improve estimates for areas that were not sampled or were poorly sampled [50].

The observed results correspond to a country in which the epidemiological transition trends in diabetes and obesity evolve over time in the population and the progressive prevalence increases first in the high-income and then in the low-income groups [51]. An epidemiological transition due to economic changes and urbanization was also observed in the South Asian population. This translates into lifestyle changes toward eating a highly refined calories and saturated fat diet, and less physical activity [52].

This study has several strengths. The CHS-2 had a high response rate (80%) among the eligible population and the sampling method was adequate to make this study representative of the core population. The survey applied robust quality control of fieldwork, laboratory measurements and analyses, in accordance with updated international standards. In addition, the analysis was based on both individual and aggregate levels in a joint model approach. Moreover, we imputed missing data, making the results less likely to be biased due to data missing at random. In addition, Bayesian hierarchical modelling was able to estimate prevalence (posterior probabilities) in provinces that were not sampled, taking information from neighbour provinces.

This study has several limitations. First, the analysis was cross-sectional and we did not adjust for all possible confounders. Also, we assessed diabetes with fasting glucose and self-reported medical diagnosis in addition to the measured fasting blood sugar to increase the sensitivity of the diagnosis (some participants with diagnosed diabetes may have normal blood sugar on the day of the exam). However, self-reported diagnosis may be subject of recall-bias. With LDL, percentage of missing data was high and therefore, the multiple imputed estimates from the Bayesian results show less precision. Furthermore, we cannot exclude selection bias in the sampling of some provinces. However, hierarchical Bayesian modelling provides precise results, in particular for areas with low sampling and missing data. This is due to the smoothing effect of neighbour provinces over the estimates.

## Conclusions

The joint model analysis presented in this study gives a good approximation of the reality for the identification of hotspots in cardiometabolic outcomes and addresses the issues of small numbers and missing data. Hotspots were mostly located in certain provinces in the centre and south/far south great regions. This is an important piece of information for local public health authorities. In addition, the results of this study show evidence of the utility of Bayesian hierarchical modelling to monitor the general population in countries with issues of access to remote areas and heterogeneous distribution of the population. The methods used in this study, which are reproducible and scalable, allow the identification of affected provinces in order to inform priority actions for cardiometabolic prevention.

Our approach is innovative compared to other spatial analytical methods as the joint model analysis makes it possible to link the individual results while taking into account the correlation between areas (aggregated level). In addition, this methodology can also deal with missing data generating predicted estimates derived from the correlation between the results.

## Supporting information

S1 Table Age and sex adjusted frequentist prevalence of cardiometabolic outcomes (diabetes, obesity, hypertension and high LDL cholesterol). (DOCX)

S2 Table Prevalence of cardiometabolic variables (posterior probabilities): Basic and further adjusted model (sensitivity analysis). (DOCX)

## References

[1] Roth GA, Johnson C, Abajobir A, Abd-Allah F, Abera SF, Abyu G, et al Global, regional, and national burden of cardiovascular diseases for 10 causes, 1990 to 2015. Journal of the American College of Cardiology. 2017;70(1):1–25. 10.1016/j.jacc.2017.04.052 28527533 PMC5491406

[2] Yeates K, Lohfeld L, Sleeth J, Morales F, Rajkotia Y, Ogedegbe O. A global perspective on cardiovascular disease in vulnerable populations. Canadian Journal of Cardiology. 2015;31(9):1081–93. 10.1016/j.cjca.2015.06.035 26321432 PMC4787293

[3] Popkin BM. Nutrition Transition and the Global Diabetes Epidemic. Current diabetes reports. 2015;15(9):64 10.1007/s11892-015-0631-4 26209940 PMC4942180

[4] Albala C, Vio F, Kain J, Uauy R. Nutrition transition in Chile: determinants and consequences. Public health nutrition. 2002;5(1a):123–8. 10.1079/PHN2001283 12027274

[5] Ministerio de Salud DdPS, Departamento de Epidemiología. Encuesta de Salud Chile 2003 [Chile Health Survey 2003]. 2003.

[6] Ministerio de Salud DdPS, Departamento de Epidemiología. Encuesta Nacional de Salud Chile 2009–2010, Resultados [National Health Survey Chile 2009–2010, Results]. 2011.

[7] Ministerio de Salud DdPS, Departamento de Epidemiología. Encuesta Nacional de Salud 2016–2017 Primeros resultados [National Health Survey 2016–2017 First results]. 2017.

[8] Clark-Núñez X. Compendio estadístico [Statistical Compendium]. Instituto Nacional de Estadísticas Chile; 2017.

[9] Matute I, Burgos S, Alfaro T. Socioeconomic status and perceived health-related quality of life in Chile. MEDICC Review. 2017;19:51–6. 10.37757/MR2017.V19.N2-3.9 34352978

[10] Subramanian S, Delgado I, Jadue L, Vega J, Kawachi I. Income inequality and health: multilevel analysis of Chilean communities. Journal of Epidemiology & Community Health. 2003;57(11):844–8. 14600107 10.1136/jech.57.11.844 PMC1732331

[11] Cabieses B, Tunstall H, Pickett K. Understanding the socioeconomic status of international immigrants in Chile through hierarchical cluster analysis: a population‐based study. International Migration. 2015;53(2):303–20.

[12] Villalobos Dintrans P. Out-of-pocket health expenditure differences in Chile: Insurance performance or selection? Health policy (Amsterdam, Netherlands). 2018;122(2):184–91. 10.1016/j.healthpol.2017.11.007 29169610

[13] Timonin S, Kontsevaya A, McKee M, Leon DA. Reducing geographic inequalities in access times for acute treatment of myocardial infarction in a large country: the example of Russia. Int J Epidemiol. 2018;47(5):1594–602. 10.1093/ije/dyy146 30085113 PMC6208271

[14] Diez Roux AV. Investigating neighborhood and area effects on health. American journal of public health. 2001;91(11):1783–9. 10.2105/ajph.91.11.1783 11684601 PMC1446876

[15] de Groot R, van den Hurk K, Schoonmade LJ, de Kort W, Brug J, Lakerveld J. Urban-rural differences in the association between blood lipids and characteristics of the built environment: a systematic review and meta-analysis. BMJ Glob Health. 2019;4(1):e001017 10.1136/bmjgh-2018-001017 30740247 PMC6347938

[16] Gaglioti AH, Xu J, Rollins L, Baltrus P, O'Connell LK, Cooper DL, et al Neighborhood Environmental Health and Premature Death From Cardiovascular Disease. Prev Chronic Dis. 2018;15:E17 10.5888/pcd15.170220 29389312 PMC5798222

[17] Di Cesare M, Bhatti Z, Soofi SB, Fortunato L, Ezzati M, Bhutta ZA. Geographical and socioeconomic inequalities in women and children's nutritional status in Pakistan in 2011: an analysis of data from a nationally representative survey. Lancet Glob Health. 2015;3(4):e229–39. 10.1016/S2214-109X(15)70001-X 25794676 PMC4365918

[18] Kirby RS, Delmelle E, Eberth JM. Advances in spatial epidemiology and geographic information systems. Annals of Epidemiology. 2017;27(1):1–9. 10.1016/j.annepidem.2016.12.001 28081893

[19] Solimano G, Mazzei M. ¿ De qué mueren los chilenos hoy?: perspectivas para el largo plazo [What do Chileans die of today?: long-term outlook]. Revista médica de Chile. 2007;135(7):932–8. 10.4067/s0034-98872007000700015 17914552

[20] Mindell JS, Moody A, Vecino-Ortiz AI, Alfaro T, Frenz P, Scholes S, et al Comparison of health examination survey methods in Brazil, Chile, Colombia, Mexico, England, Scotland and the USA. American journal of epidemiology. 2017:kwx045. 10.1093/aje/kwx045 PMC5860286 28486584

[21] Albers C. Coberturas SIG de Chile [Available from: http://labgeo.ufro.cl/catalogos/chile.html.

[22] Expert Committee on the D, Classification of Diabetes M. Report of the expert committee on the diagnosis and classification of diabetes mellitus. Diabetes Care. 2003;26 Suppl 1(suppl 1):S5–20. 12502614 10.2337/diacare.26.2007.s5

[23] Organization WH. WHO Expert Committee on Physical Status: the use and interpretation of anthropometry. Geneva: World Health Organization; 1995 WHO Technical Report Series. 1995;854. 8594834

[24] The sixth report of the Joint National Committee on prevention, detection, evaluation, and treatment of high blood pressure. Archives of internal medicine. 1997;157(21):2413–46. 10.1001/archinte.157.21.2413 9385294

[25] Ardern CI, Katzmarzyk PT, Janssen I, Church TS, Blair SN. Revised Adult Treatment Panel III guidelines and cardiovascular disease mortality in men attending a preventive medical clinic. Circulation. 2005;112(10):1478–85. 10.1161/CIRCULATIONAHA.105.548198 16129792

[26] Tanner MA, Wong WH. The Calculation of Posterior Distributions by Data Augmentation. Journal of the American Statistical Association. 1987;82(398):528–40.

[27] Lawson A, Schritz A, Villarroel L, Aguayo GA. Multi-Scale Multivariate Models for Small Area Health Survey Data: A Chilean Example. International journal of environmental research and public health. 2020;17(5):E1682 10.3390/ijerph17051682 32150815 PMC7084380

[28] Aregay M, Lawson AB, Faes C, Kirby RS. Bayesian multi-scale modeling for aggregated disease mapping data. Statistical methods in medical research. 2015. 10.1177/0962280215607546 PMC5376246 26420779

[29] Vandendijck Y, Faes C, Kirby RS, Lawson A, Hens N. Model-based inference for small area estimation with sampling weights. Spatial Statistics. 2016;18:455–73. 10.1016/j.spasta.2016.09.004 28989860 PMC5627524

[30] Watjou K, Faes C, Lawson A, Kirby RS, Aregay M, Carroll R, et al Spatial small area smoothing models for handling survey data with nonresponse. Statistics in Medicine. 2017;36(23):3708–45. 10.1002/sim.7369 28670709 PMC5585068

[31] Carroll R, Lawson AB, Faes C, Kirby RS, Aregay M, Watjou K. Extensions to Multivariate Space Time Mixture Modeling of Small Area Cancer Data. International journal of environmental research and public health. 2017;14(5). 10.3390/ijerph14050503 PMC5451954 28486417

[32] Lawson A, Schritz A, Villarroel L, Aguayo G. Multi-Scale Multivariate Models for Small Area Health Survey Data: A Chilean Example. International journal of environmental research and public health. 2020;17(5):1682. 10.3390/ijerph17051682 PMC7084380 32150815

[33] Lawson AB, Rotejanaprasert C. Childhood brain cancer in Florida: a Bayesian clustering approach. Statistics and Public Policy. 2014;1(1):99–107.

[34] Lunn DJ, Thomas A, Best N, Spiegelhalter D. WinBUGS-a Bayesian modelling framework: concepts, structure, and extensibility. Statistics and computing. 2000;10(4):325–37.

[35] Sturtz S, Ligges U, Gelman AE. R2WinBUGS: a package for running WinBUGS from R. 2005.

[36] Tennekes M. tmap: Thematic Maps in R. J Stat Softw. 2018;84:1–39. 10.18637/jss.v084.i01 30450020 PMC6238955

[37] Ripley BD. Modern applied statistics with S: Springer; 2002.

[38] Collaboration NRF. Worldwide trends in diabetes since 1980: a pooled analysis of 751 population-based studies with 4· 4 million participants. The Lancet. 2016;387(10027):1513–30. 10.1016/S0140-6736(16)00618-8 PMC5081106 27061677

[39] Ng M, Fleming T, Robinson M, Thomson B, Graetz N, Margono C, et al Global, regional, and national prevalence of overweight and obesity in children and adults during 1980–2013: a systematic analysis for the Global Burden of Disease Study 2013. Lancet (London, England). 2014;384(9945):766–81. 10.1016/S0140-6736(14)60460-8 24880830 PMC4624264

[40] Mills KT, Bundy JD, Kelly TN, Reed JE, Kearney PM, Reynolds K, et al Global Disparities of Hypertension Prevalence and Control: A Systematic Analysis of Population-Based Studies From 90 Countries. Circulation. 2016;134(6):441–50. 10.1161/CIRCULATIONAHA.115.018912 27502908 PMC4979614

[41] Kuklina EV, Carroll MD, Shaw KM, Hirsch R. Trends in high LDL cholesterol, cholesterol-lowering medication use, and dietary saturated-fat intake: United States, 1976–2010. NCHS data brief. 2013(117):1. PMC4401469 23759124

[42] Gattini C, Chávez C, Albers D. Comunas de Chile, según nivel socio-económico, de salud y desarrollo humano. Revisión 2013 [Communes of Chile, according to socio-economic level, health and human development. 2013 revision]. Documento de Serie Técnica del Observatorio Chileno de Salud Pública. 2014;3.

[43] Ferreccio C, Roa JC, Bambs C, Vives A, Corvalan AH, Cortes S, et al Study protocol for the Maule Cohort (MAUCO) of chronic diseases, Chile 2014–2024. BMC public health. 2016;16:122 10.1186/s12889-015-2454-2 26847446 PMC4743396

[44] Connolly V, Unwin N, Sherriff P, Bilous R, Kelly W. Diabetes prevalence and socioeconomic status: a population based study showing increased prevalence of type 2 diabetes mellitus in deprived areas. Journal of Epidemiology & Community Health. 2000;54(3):173–7. 10746110 10.1136/jech.54.3.173 PMC1731634

[45] Mokdad AH, Ford ES, Bowman BA, Dietz WH, Vinicor F, Bales VS, et al Prevalence of obesity, diabetes, and obesity-related health risk factors, 2001. Jama. 2003;289(1):76–9. 10.1001/jama.289.1.76 12503980

[46] Ramachandran A, Mary S, Yamuna A, Murugesan N, Snehalatha C. High prevalence of diabetes and cardiovascular risk factors associated with urbanization in India. Diabetes Care. 2008;31(5):893–8. 10.2337/dc07-1207 18310309

[47] Thornton LE, Bentley RJ, Kavanagh AM. Individual and area-level socioeconomic associations with fast food purchasing. Journal of Epidemiology and Community Health. 2011;65(10):873–80. 10.1136/jech.2009.099614 20889585

[48] Ruiz-Castell M, Muckle G, Dewailly É, Jacobson JL, Jacobson SW, Ayotte P, et al Household crowding and food insecurity among Inuit families with school-aged children in the Canadian Arctic. 2015;105(3):e122–e32. 10.2105/AJPH.2014.302290 25602890 PMC4330833

[49] Leng B, Jin Y, Li G, Chen L, Jin N. Socioeconomic status and hypertension: a meta-analysis. Journal of hypertension. 2015;33(2):221–9. 10.1097/HJH.0000000000000428 25479029

[50] Pringle D. Mapping disease risk estimates based on small numbers: an assessment of empirical Bayes techniques. Economic and social review. 1996;27:341–64.

[51] Jaacks LM, Vandevijvere S, Pan A, McGowan CJ, Wallace C, Imamura F, et al The obesity transition: stages of the global epidemic. The Lancet Diabetes & Endocrinology. 2019. 10.1016/S2213-8587(19)30026-9 PMC7360432 30704950

[52] Misra A, Soares MJ, Mohan V, Anoop S, Abhishek V, Vaidya R, et al Body fat, metabolic syndrome and hyperglycemia in South Asians. Journal of diabetes and its complications. 2018;32(11):1068–75. 10.1016/j.jdiacomp.2018.08.001 30115487

