# Supplementary material for: Identifying hotspots of cardiometabolic outcomes based on a Bayesian approach: The example of Chile
Source: PLoS One. 2020 Jun 22;15(6):e0235009. doi: 10.1371/journal.pone.0235009 (PMC7307745; doi:10.1371/journal.pone.0235009)
Supplement: S1 Table — (DOCX) [file pone.0235009.s001.docx]

S1 Table. Age and sex adjusted frequentist prevalence of cardiometabolic outcomes (diabetes, obesity, hypertension and high LDL cholesterol)

| Province (number) | Sampled | % (95% CI) | % (95% CI) | % (95% CI) | % (95% CI) |
| --- | --- | --- | --- | --- | --- |
| North |  |  |  |  |  |
| Parinacota (1) | 0 | NA | NA | NA | NA |
| Arica (2) | 280 | 10 (9.3, 11) | 28 (27, 29) | 30 (26, 33) | 12 (11, 12) |
| Iquique (3) | 253 | 10 (9, 11) | 23 (23, 24) | 31 (27, 34) | 14 (13, 15) |
| Tamarugal (4) | 21 | 9.7 (6.3, 13) | 30 (27, 33) | 33 (22, 45) | 12 (9.9, 15) |
| Tocopilla (5) | 31 | 9.4 (6.5, 12) | 31, (29, 33) | 33 (23, 43) | 14 (12, 16) |
| El Loa (6) | 78 | 7.5 (5.9, 9.1) | 27 (25, 28) | 24 (18, 30) | 11 (9.3, 12) |
| Antofagasta (7) | 176 | 9.2 (8.1, 10) | 30 (29, 31) | 31 (27, 35) | 12 (11, 13) |
| Chañaral (8) | 0 | NA | NA | NA | NA |
| Copiapó (9) | 214 | 10 (9.1, 11) | 31 (30, 32) | 32 (29, 36) | 14 (13, 15) |
| Huasco (10) | 75 | 12 (9.9, 15) | 29 (27, 31) | 40 (33, 48) | 14 (13, 16) |
| Elqui (11) | 171 | 9.7 (8.3, 11) | 31 (30, 32) | 33 (29, 37) | 10 (9.3, 12) |
| Limarí (12) | 68 | 12 (9.7, 14) | 25 (24, 27) | 41 (34, 48) | 15 (13, 17) |
| Choapa (13) | 43 | 12 (8.4, 15) | 26 (23, 28) | 33 (22, 43) | 14 (11, 17) |
| Center |  |  |  |  |  |
| San Antonio (14) | 31 | 9.4 (7, 12) | 29 (26, 32) | 32 (23, 41) | 12 (10.0, 14) |
| Petorca (15) | 15 | 11 (6.6, 15) | 27 (23, 32) | 40 (24, 56) | 13 (10, 16) |
| Valparaíso (16) | 171 | 12 (10, 13) | 27 (26, 28) | 42 (38, 47) | 14 (13, 16) |
| Quillota (17) | 45 | 13 (10, 16) | 31 (28, 34) | 44 (34, 53) | 12 (9.4, 14) |
| Los Andes (18) | 22 | 11 (6.4, 15) | 29 (26, 33) | 33 (21, 46) | 14 (7.3, 21) |
| San Felipe de Aconcagua (19) | 26 | 13 (9.3, 16) | 33 (30, 36) | 47 (35, 60) | 13 (11, 16) |
| Chacabuco (20) | 12 | 5.8 (2.9, 8.8) | 26 (22, 31) | 19 (5.1, 32) | 11 (5.9, 15) |
| Santiago (21) | 636 | 10 (9.5, 11) | 25 (24, 25) | 32 (30, 35) | 9.1 (8.6, 9.5) |
| Melipilla (22) | 21 | 12 (7.4, 16) | 27 (24, 31) | 42 (30, 54) | 8.6 (7.2, 10.0) |
| Talagante (23) | 17 | 7.2 (4.3, 10) | 24 (20, 28) | 25 (13, 37) | 9.8 (6.9, 13) |
| Maipo (24) | 43 | 10 (7.8, 13) | 31 (28, 33) | 35 (26, 44) | 10 (8.5, 12) |
| Cordillera (25) | 73 | 9.1 (7.1, 11) | 26 (24, 27) | 29 (22, 35) | 10 (8.6, 12) |
| Cardenal Caro (26) | 0 | NA | NA | NA | NA |
| Cachapoal (27) | 202 | 12 (10, 13) | 28 (27, 29) | 41 (37, 45) | 11 (9.7, 12) |
| Colchagua (28) | 97 | 14 (12, 16) | 25 (24, 27) | 51 (45, 58) | 12 (10, 13) |
| Cauquenes (29) | 15 | 15 (10, 20) | 32 (28, 35) | 53 (37, 69) | 17 (14, 20) |
| Curicó (30) | 76 | 12 (10, 15) | 30 (28, 32) | 41 (34, 49) | 13 (11, 14) |
| Linares (31) | 91 | 9.6 (7.9, 11) | 33 (31, 35) | 33 (27, 39) | 13 (12, 15) |
| Talca (32) | 122 | 8.8 (7.4, 10) | 29 (27, 30) | 31 (26, 37) | 11 (10, 12) |
| Arauco (33) | 47 | 10 (7.9, 13) | 32 (29, 34) | 38 (30, 47) | 13 (11, 15) |
| Concepción (34) | 116 | 10 (8.9, 12) | 28 (26, 29) | 38 (33, 44) | 14 (13, 16) |
| Ñuble (35) | 51 | 9.2 (7, 12) | 25 (23, 27) | 29 (21, 37) | 9.0 (7.1, 11) |
| Biobío (36) | 43 | 12 (8.6, 15) | 34 (31, 37) | 42 (32, 51) | 13 (10, 15) |
| South |  |  |  |  |  |
| Malleco (37) | 55 | 13 (10, 15) | 31 (29, 33) | 48 (41, 56) | 13 (12, 15) |
| Cautín (38) | 232 | 11 (9.7, 12) | 34 (33, 35) | 40 (36, 44) | 13 (12, 14) |
| Ranco (39) | 69 | 12 (9.6, 15) | 31 (29, 33) | 42 (34, 49) | 15 (13, 17) |
| Valdivia (40) | 213 | 11 (10, 13) | 35 (33, 36) | 37 (33, 41) | 12 (11, 12) |
| Chiloé (41) | 68 | 11 (8.6, 12) | 32 (30, 34) | 39 (32, 46) | 17 (14, 19) |
| Llanquihue (42) | 131 | 12 (11, 14) | 29 (28, 31) | 41 (35, 46) | 13 (12, 14) |
| Palena (43) | 0 | NA | NA | NA | NA |
| Osorno (44) | 89 | 12 (9.5, 14) | 33 (31, 35) | 39 (33, 46) | 14 (13, 16) |
| Far south |  |  |  |  |  |
| Coyhaique (45) | 160 | 9.4 (8.3, 11) | 31 (29, 32) | 35 (30, 39) | 16 (15, 18) |
| General Carrera (46) | 85 | 7.7 (6.1, 9.3) | 33 (32, 35) | 25 (20, 30) | 12 (11, 13) |
| Aysén (47) | 0 | NA | NA | NA | NA |
| Capitan Prat (48) | 0 | NA | NA | NA | NA |
| Antártica Chilena (49) | 0 | NA | NA | NA | NA |
| Ultima Esperanza (50) | 47 | 13 (9.8, 16) | 32 (30, 35) | 45 (36, 54) | 16 (14, 19) |
| Magallanes (51) | 236 | 11 (10, 12) | 35 (34, 36) | 36 (32, 40) | 15 (14, 16) |
| Tierra del Fuego (52) | 11 | 7.8 (4.6, 11) | 27 (24, 30) | 28 (14, 42) | 12 (9.5, 15) |

Abbreviations: 95% CI: 95% confidence interval; NA: non applicable. *number of participants sampled in that province.
