# Supplementary material for: Identifying hotspots of cardiometabolic outcomes based on a Bayesian approach: The example of Chile
Source: PLoS One. 2020 Jun 22;15(6):e0235009. doi: 10.1371/journal.pone.0235009 (PMC7307745; doi:10.1371/journal.pone.0235009)
Supplement: S2 Table — (DOCX) [file pone.0235009.s002.docx]

S2 Table. Prevalence of cardiometabolic variables (posterior probabilities): basic model and further adjusted model (sensitivity analysis)

|  | Diabetes | | Obesity | | Hypertension | | High LDL cholesterol | |
| --- | --- | --- | --- | --- | --- | --- | --- | --- |
|  | Posterior probability (% (95% Credible Intervals)) | | | | | | | |
| . | Basic model | Sensitivity analysis | Basic model | Sensitivity analysis | Basic model | Sensitivity analysis | Basic model | Sensitivity analysis |
| North |  |  |  |  |  |  |  |  |
| Parinacota (1) | 13 (4.7, 28) | 12 (1.8, 36) | 27 (14, 45) | 34 (11, 70) | 31 (14, 56) | 41 (15, 75) | 17 (2.0, 52) | 13 (1.0, 47) |
| Arica (2) | 11 (8.4, 14) | 9.0 (7.1, 11) | 28 (24, 32) | 27 (23, 32) | 26 (22, 30) | 30 (25, 33) | 11 (7.4, 16) | 12 (8.0, 16) |
| Iquique (3) | 11 (8.7, 14) | 10 (7.9, 13) | 20 (17, 24) | 24 (19, 28) | 28 (24, 32) | 30 (26, 34) | 16 (11, 21) | 15 (11, 20) |
| Tamarugal (4) | 12 (8.4, 18) | 13 (8.0, 19) | 25 (17, 34) | 23 (17, 32) | 29 (23, 37) | 30 (24, 37) | 22 (11, 38) | 18 (9.5, 31) |
| Tocopilla (5) | 10 (7.1, 15) | 11 (7.6, 15) | 30 (22, 40) | 28 (21, 35) | 28 (22, 36) | 29 (24, 34) | 20 (10, 35) | 16 (9.1, 26) |
| El Loa (6) | 9 (6.1, 13) | 8.1 (5.2, 12) | 27 (20, 34) | 29 (22, 36) | 22 (17, 28) | 20 (15, 25) | 13 (6.5, 22) | 14 (7.7, 23) |
| Antofagasta (7) | 11 (7.9, 14) | 9.8 (7.8, 12) | 29 (24, 35) | 28 (24, 33) | 28 (23, 33) | 27 (24, 31) | 13 (8.2, 19) | 13 (8.8, 18) |
| Chañaral (8) | 13 (4.6, 27) | 12 (1.7, 37) | 28 (14, 46) | 36 (11, 71) | 34 (16, 59) | 41 (15, 75) | 16 (1.8, 52) | 13 (1.0, 48) |
| Copiapó (9) | 10 (7.7, 13) | 10 (8.3, 13) | 31 (27, 36) | 31 (26, 36) | 31 (27, 36) | 32 (29, 37) | 15 (10, 21) | 15 (10, 20) |
| Huasco (10) | 12 (8.3, 16) | 12 (8.8, 15) | 25 (19, 32) | 27 (21, 33) | 38 (32, 45) | 40 (34, 45) | 15 (8.5, 24) | 14 (8.8, 21) |
| Elqui (11) | 11 (8.0, 14) | 10 (7.9, 13) | 31 (26, 37) | 30 (25, 35) | 33 (28, 38) | 31 (27, 35) | 8.9 (4.8, 14) | 10 (6.1, 15) |
| Limarí (12) | 13 (9.2, 18) | 10 (7.0, 13) | 20 (14, 26) | 23 (17, 30) | 39 (33, 46) | 40 (34, 46) | 14 (7.5, 24) | 13 (7.7, 21) |
| Choapa (13) | 9.7 (5.9, 13) | 10 (7.2, 13) | 21 (14, 29) | 24 (18, 30) | 34 (27, 41) | 35 (30, 39) | 16.8 (4, 27) | 13 (8.0, 21) |
| Center |  |  |  |  |  |  |  |  |
| San Antonio (14) | 9.4 (5.7, 14) | 9.7 (6.3, 14) | 25 (17, 34) | 21 (15, 29) | 33 (25, 41) | 33 (27, 40) | 15.6 (6, 29) | 11 (5.4, 20) |
| Petorca (15) | 13 (8.2, 20) | 14 (7.6, 23) | 26 (17, 37) | 33 (22, 47) | 43 (34, 53) | 44 (33, 56) | 9.5 (3.6, 20) | 10 (3.6, 22) |
| Valparaíso (16) | 12 (8.6, 15) | 13 (10, 17) | 24 (20, 29) | 24 (20, 29) | 44 (39, 49) | 41 (36, 46) | 13 (7.9, 20) | 12 (7.5, 17) |
| Quillota (17) | 12 (8.5, 17) | 12 (8.7, 17) | 29 (22, 38) | 30 (23, 37) | 44 (37, 51) | 47 (40, 53) | 11 (4.8, 20) | 11 (5.8, 19) |
| Los Andes (18) | 11 (7.4, 16) | 11 (7.4, 16) | 26 (18, 35) | 25 (18, 32) | 34 (27, 41) | 37 (30, 43) | 12 (5.2, 23) | 11 (5.6, 19) |
| San Felipe de Aconcagua (19) | 16 (11, 24) | 17 (10, 25) | 32 (22, 43) | 32 (23, 42) | 50 (42, 59) | 51 (43, 60) | 8.2 (3.0, 16) | 9.9 (4.0, 18) |
| Chacabuco (20) | 6.2 (3.1, 11) | 6.2 (3.2, 11) | 31 (20, 44) | 34 (24, 46) | 20 (13, 28) | 19 (13, 26) | 9.0.2 (7, 21) | 9.7 (3.5, 20) |
| Santiago (21) | 9.9 (8.0, 12) | 9.5 (7.8, 11) | 24 (21, 27) | 24 (21, 27) | 32 (29, 35) | 33 (30, 36) | 7.7.5 (5, 10) | 8.6 (6.2, 11) |
| Melipilla (22) | 14 (9.3, 22) | 11 (7.0, 17) | 25 (17, 35) | 25 (17, 34) | 45 (37, 56) | 51 (42, 59) | 8.4 (3.0, 17) | 6.2 (2.3, 13) |
| Talagante (23) | 11 (4.0, 23) | 11 (3.8, 24) | 28 (15, 44) | 28 (14, 44) | 25 (14, 40) | 26 (14, 41) | 12 (2.3, 32) | 8.8 (1.5, 25) |
| Maipo (24) | 10 (6.7, 14) | 12 (8.4, 16) | 31 (23, 41) | 26 (20, 34) | 35 (29, 43) | 36 (30, 42) | 8.2 (3.4, 15) | 9.1 (4.6, 15) |
| Cordillera (25) | 8.7 (5.8, 12) | 8.2 (5.6, 11) | 26 (19, 32) | 27 (21, 33) | 29 (23, 35) | 30 (25, 35) | 7.5.3 (3, 14) | 8.6 (4.5, 14) |
| Cardenal Caro (26) | 12 (4.3, 27) | 12 (1.9, 38) | 27 (14, 45) | 36 (12, 72) | 40 (20, 65) | 41 (15, 75) | 10 (1.0, 34) | 13 (1.0, 45) |
| Cachapoal (27) | 11 (8.0, 13) | 10 (8.5, 13) | 26 (21, 30) | 26 (22, 31) | 42 (38, 47) | 40 (36, 44) | 7.5 (4.0, 12) | 10 (6.1, 14) |
| Colchagua (28) | 13 (9.0, 17) | 11 (7.8, 14) | 21 (15, 27) | 25 (19, 31) | 54 (48, 61) | 52 (47, 58) | 5.2 (1.9, 10) | 8.3 (3.9, 14) |
| Cauquenes (29) | 16 (9.8, 24) | 14 (8.2, 22) | 27 (18, 39) | 25 (16, 35) | 57 (46, 67) | 59 (49, 68) | 20 (9.2, 37) | 14 (6.5, 27) |
| Curicó (30) | 14 (10.2, 19) | 13 (9.2, 18) | 29 (22, 36) | 31 (24, 38) | 44 (37, 50) | 45 (38, 52) | 8.7 (4.3, 14) | 10 (5.5, 16) |
| Linares (31) | 9.8 (7.1, 13) | 9.1 (6.5, 12) | 35 (28, 42) | 32 (26, 39) | 35 (29, 41) | 35 (29, 40) | 13.1.7 (6, 20) | 13 (7.6, 19) |
| Talca (32) | 7.8 (5.2, 11) | 8.3 (5.9, 11) | 30 (24, 36) | 32 (26, 39) | 33 (28, 39) | 31 (26, 36) | 11.6 (6, 16) | 11 (7.1, 16) |
| Arauco (33) | 12 (8.6, 17) | 12 (8.1, 16) | 36 (28, 45) | 36 (29, 44) | 43 (36, 51) | 42 (36, 48) | 13 (7.2, 21) | 12 (6.8, 18) |
| Concepción (34) | 13 (9.5, 18) | 14 (10, 17) | 28 (22, 34) | 29 (23, 35) | 40 (34, 46) | 37 (32, 42) | 16 (9.6, 23) | 13 (7.7, 20) |
| Ñuble (35) | 8.7 (5.0, 13) | 8.2 (5.1, 12) | 26 (19, 35) | 29 (21, 36) | 32 (25, 39) | 35 (29, 42) | 10.4 (0, 19) | 8.0 (3.6, 15) |
| Biobío (36) | 11 (6.9, 15) | 13 (9.3, 17) | 38 (30, 48) | 31 (25, 40) | 46 (39, 55) | 44 (39, 50) | 13 (6.4, 22) | 11 (6.4, 18) |
| South |  |  |  |  |  |  |  |  |
| Malleco (37) | 12 (7.6, 18) | 11 (6.5, 16) | 33 (25, 42) | 32 (24, 41) | 56 (47, 64) | 53 (46, 61) | 8.8 (3.6, 16) | 9.8 (4.4, 17) |
| Cautín (38) | 12 (9.8, 15) | 13 (10, 15) | 36 (31, 41) | 34 (30, 39) | 42 (37, 47) | 39 (36, 44) | 12 (7.7, 17) | 12 (8.6, 17) |
| Ranco (39) | 11 (7.9, 15) | 11 (8.4, 14) | 33 (26, 41) | 33 (27, 40) | 43 (37, 50) | 42 (37, 47) | 15 (8.2, 23) | 14 (9.0, 21) |
| Valdivia (40) | 11 (8.2, 14) | 11 (8.6, 14) | 36 (31, 42) | 34 (29, 40) | 37 (32, 42) | 38 (33, 42) | 11 (6.7, 16) | 13 (8.3, 18) |
| Chiloé (41) | 9.9 (6.6, 13) | 12 (8.3, 17) | 31 (25, 39) | 32 (25, 39) | 38 (33, 45) | 37 (31, 44) | 21 (13, 33) | 16 (9.8, 26) |
| Llanquihue (42) | 11 (7.9, 14) | 12 (9.4, 15) | 29 (24, 35) | 30 (25, 36) | 40 (34, 46) | 40 (36, 45) | 14 (8.4, 20) | 14 (9.4, 20) |
| Palena (43) | 10 (3.6, 23) | 13 (2.1, 40) | 34 (19, 52) | 41 (13, 76) | 38 (19, 63) | 41 (15, 75) | 20 (2.8, 58) | 17 (1.3, 56) |
| Osorno (44) | 10 (7.0, 14) | 12 (8.6, 15) | 37 (30, 44) | 37 (30, 43) | 41 (35, 48) | 43 (38, 49) | 14 (8.2, 22) | 14 (8.3, 20) |
| Far south |  |  |  |  |  |  |  |  |
| Coyhaique (45) | 7.9 (5.3, 11) | 9.7 (7.5, 12) | 32 (27, 38) | 34 (28, 39) | 35 (30, 41) | 32 (28, 37) | 22 (14, 32) | 19 (13, 28) |
| General Carrera (46) | 5.8 (3.0, 9.7) | 7.7 (4.3, 12) | 37 (29, 46) | 37 (29, 46) | 26 (19, 33) | 25 (19, 32) | 17.9 (8, 26) | 16 (9.2, 25) |
| Aysén (47) | 10 (3.5, 23) | 13 (2.1, 40) | 34 (19, 54) | 41 (14, 75) | 37 (18, 62) | 42 (16, 76) | 22 (2.9, 62) | 17 (1.5, 56) |
| Capitan Prat (48) | 10 (3.6, 23) | 13 (2.1, 40) | 35 (19, 54) | 42 (15, 77) | 37 (18, 61) | 41 (15, 75) | 22 (3.0, 63) | 17 (1.5, 60) |
| Antártica Chilena (49) | 12 (0.8, 37) | 12 (1.4, 40) | 41 (14, 71) | 34 (6, 76) | 22 (4, 54) | 30 (3.2, 74) | 15 (0.4, 62) | 12 (0.1, 56) |
| Ultima Esperanza (50) | 10 (6.5, 15) | 11 (7.6, 16) | 36 (27, 45) | 35 (28, 43) | 45 (37, 54) | 46 (40, 53) | 24 (13, 37) | 18 (10, 29) |
| Magallanes (51) | 10 (7.6, 13) | 12 (9.1, 15) | 38 (33, 43) | 36 (31, 42) | 35 (30, 40) | 36 (31, 40) | 17 (12, 23) | 17 (12, 23) |
| Tierra del Fuego (52) | 10 (0.9, 29) | 13 (4.1, 27) | 40 (19, 63) | 37 (13, 65) | 18 (5, 38) | 19 (2.5, 45) | 12 (0.9, 37) | 12 (0.4, 38) |

Basic model: fixed effects are sample weight, age, sex and age-sex interaction. Sensitivity analysis further adjusted by education and income.
